# Vulpinic Acid Controls Stem Cell Fate toward Osteogenesis and Adipogenesis

**DOI:** 10.3390/genes11010018

**Published:** 2019-12-23

**Authors:** Sang Ah Yi, Ki Hong Nam, Sil Kim, Hae Min So, Rhim Ryoo, Jeung-Whan Han, Ki Hyun Kim, Jaecheol Lee

**Affiliations:** 1 School of Pharmacy, Sungkyunkwan University, Suwon 16419, Korea; angelna1023@hanmail.net (S.A.Y.); nam6422@hanmail.net (K.H.N.); malin_1272@naver.com (S.K.); haemi9312@gmail.com (H.M.S.); jhhan551@skku.edu (J.-W.H.); 2 Special Forest Products Division, Forest Bioresources Department, National Institute of Forest Science, Suwon 16631, Korea; rryoo@korea.kr; 3 Imnewrun Biosciences Inc., Suwon 16419, Korea

**Keywords:** vulpinic acid, cell fate, acetyl tubulin, acetyl H3, osteogenesis, adipogenesis, hypertrophy

## Abstract

Vulpinic acid, a naturally occurring methyl ester of pulvinic acid, has been reported to exert anti-fungal, anti-cancer, and anti-oxidative effects. However, its metabolic action has not been implicated yet. Here, we show that vulpinic acid derived from a mushroom, *Pulveroboletus ravenelii* controls the cell fate of mesenchymal stem cells and preadipocytes by inducing the acetylation of histone H3 and α-tubulin, respectively. The treatment of 10T1/2 mesenchymal stem cells with vulpinic acid increased the expression of Wnt6, Wnt10a, and Wnt10b, which led to osteogenesis inhibiting the adipogenic lineage commitment, through the upregulation of H3 acetylation. By contrast, treatment with vulpinic acid promoted the terminal differentiation of 3T3-L1 preadipocytes into mature adipocytes. In this process, the increase in acetylated tubulin was accompanied, while acetylated H3 was not altered. As excessive generation of adipocytes occurs, the accumulation of lipid drops was not concentrated, but dispersed into a number of adipocytes. Consistently, the expressions of lipolytic genes were upregulated and inflammatory factors were downregulated in adipocytes exposed to vulpinic acid during adipogenesis. These findings reveal the multiple actions of vulpinic acid in two stages of differentiation, promoting the osteogenesis of mesenchymal stem cells and decreasing hypertrophic adipocytes, which can provide experimental evidence for the novel metabolic advantages of vulpinic acid.

## 1. Introduction

Mesenchymal stem cells (MSCs), which are present in bone marrow and soft tissues, have the capacity to differentiate into diverse stromal lineages including osteogenic (bone), adipogenic (fat), myogenic (muscle), and chondrogenic (cartilage) lineages [1,2]. The differentiation of MSCs to stromal cells is achieved by several biological processes, involving clonal expansion, morphological changes, and the epigenetic regulation of lineage- and stage-specific markers [3]. Mechanisms underlying the control of the MSC differentiation process have been extensively studied, because keeping the balance between these cell lineages is crucial to maintaining metabolic homeostasis [2,3].

The determination of MSC fate requires the selective activation or repression of master regulatory genes which are specific for respective lineages, such as peroxisome proliferator-activated receptor (PPAR)γ for adipogenesis, runt-related transcription factor 2 (Runx2) for osteogenesis, and Sox9 for chondrogenesis [4]. A complex network of histone modifiers modulates the expression of the lineage-specific transcription programs. Enhancer of zeste homolog 2 (EZH2)-mediated trimethylation at lysine 27 of histone H3 (H3K27me3) has been reported to promote adipogenesis while inhibiting osteogenesis by enhancing PPARγ and suppressing Runx2, which were reversed by lysine-specific demethylase 6A (KDM6A), a demethylase of H3K27me3 [5,6]. The reciprocal actions of histone acetyltransferases (HATs) and histone deacetylases (HDACs) during MSC differentiation have been also reported [7,8,9]. While HATs p300 and CREB binding protein (CBP) promote adipogenesis through the interaction with PPARγ [7], class I HDACs disturb adipogenesis by occupying the promoter of CCAAT/enhancer binding protein (C/EBP)α gene [8]. On the other hand, HDAC6 is known to promote adipogenesis and to suppress osteogenic differentiation from MSCs [9]. During the control of lineage-associated genes, HATs and HDACs compete for the same promoter region of the genes to precisely adjust the level of histone acetylation [4].

Unlike MSCs with differentiation potential to multi-lineage cells, unipotent preadipocytes, which are committed from MSCs, have a limited capacity to differentiate into only adipocytes. The maturation of preadipocytes requires the reorganization of the cytoskeleton as well as the epigenetic regulation of the adipogenic genes, because mature adipocytes are occupied by lipid droplets with other organelles located at the periphery of the cell body [10]. Indeed, the acetylation of α-tubulin has been observed during adipogenesis from 3T3-L1 preadipocytes, contributing to microtubule-based cytoskeletal remodeling [11]. This morphological transition also requires coordination between acetyltransferase (MEC-17) and deacetylases (SIRT2 and HDAC6) [11]. The coordinated upregulation of α-tubulin acetylation should occur in a timely manner, in order to accelerate maturation toward adipocytes [11]. However, the chemicals that control cytoskeletal alterations during adipogenesis have been studied relatively less than the drugs that induce epigenetic alterations [12].

Recently, vulpinic acid, one of the lichen secondary metabolites, has emerged as a potential therapeutic substance. Vulpinic acid exhibits antibiotic activities against bacteria [13] and fungi [14]. Moreover, the anti-cancer effects of vulpinic acid have been reported, which includes anti-angiogenesis, anti-proliferative properties, and cytotoxicity to human cancer cells [15,16,17]. The protective effects of vulpinic acid against oxidative stress in endothelial cells were discovered additionally, suggesting it as a drug candidate to treat atherosclerosis [18]. Despite these numerous studies of the pharmacological actions of vulpinic acid, its metabolic effects have not yet been clearly defined.

In our previous study, vulpinic acid was isolated as a main component of the mushroom *Pulveroboletus ravenelii* [16], and in this study, we identified that vulpinic acid controls stem cell lineage specification through epigenetic and mechanistic changes. Vulpinic acid enhanced the osteogenic properties of mesenchymal stem cells by increasing H3 acetylation on the promoter regions of the *Wnt* genes and the osteogenic gene, *Runx2*. However, treatment with vulpinic acid on unipotent preadipocytes, which were already committed, enhanced de novo generations of mature adipocytes, increasing the acetylation of tubulin. As the number of adipocytes increased, the unnecessary hypertrophy of adipocytes to store lipid was suppressed. Moreover, vulpinic acid-treated adipocytes showed healthy features, such as an elevated expression of lipolytic genes and decreased expression of inflammatory genes. Our data demonstrate that vulpinic acid exerts beneficial effects against metabolic disorders regulating fate determination of mesenchymal stem cells and adipocyte precursor cells through epigenetic and cytoskeletal remodeling.

## 2. Materials and Methods

### 2.1. Cell Culture and Differentiation

Both the 10T1/2 mesenchymal stem cells and the 3T3-L1 preadipocytes were plated on 6 well plates (3 × 10^4^ cells per well). The 10T1/2 cells were grown in Dulbecco’s modified eagle medium (DMEM) with 10% fetal bovine serum (FBS) and 1% penicillin—streptomycin (P/S). For adipogenic commitment to pre-adipocytes, 10T1/2 cells were incubated for 4 days in DMEM with 10% FBS, 1% penicillin—streptomycin (P/S) and 10 ng/mL of bone morphogenetic protein 4 (BMP4). For osteogenic commitment to osteoblasts, 10T1/2 cells were incubated for 3 days in DMEM with 10% FBS, 1% P/S and 50 ng/mL of BMP2. 3T3-L1 preadipocytes were grown in DMEM with 10% bovine calf serum (BCS) and 1% P/S. When the confluency of 3T3-L1 reached 80%, the terminal differentiation to adipocytes was started. For the terminal differentiation of 3T3-L1 cells to adipocytes, the cells were incubated in DMEM with 10% FBS, 1% P/S, 0.5 mM of 3-isobuyl-1-methylxanthine (IBMX), 1 μM of dexamethasone, and 1 μg/mL of insulin for 2 days. Then, the media was replaced every other day with DMEM containing 10% FBS, 1% P/S, and 1 μg/mL of insulin. The total period of incubation time was 8 days, in which the lipid droplets were observed through a microscope.

### 2.2. Extraction and Isolation of Vulpinic Acid

Vulpinic acid was extracted and isolated as previously demonstrated [16]. Dried fruiting bodies of *P. ravenelii* were extracted with 80% aqueous methanol (MeOH) three times at room temperature and filtered. Then, the obtained extract of the filtrate was solvent-partitioned, and vulpinic acid was isolated from the hexane-soluble fraction using repeated column chromatography and high-performance liquid chromatography (HPLC) purification with a Phenomenex Luna column (Phenyl-hexyl, 250 × 10.0 mm, 5 μm), using a gradient system of acetonitrile–water (4:6 to 1:0). Vulpinic acid was identified by comparison of its nuclear magnetic resonance (NMR) spectroscopic data with reported values and liquid chromatography–mass spectrometry (LC/MS) analysis [16].

### 2.3. Immunoblotting

The proteins in the cells were extracted with Pro-Prep (Intron Biotechnology, Seongnam, Korea) and centrifuged after sonication. A total of 20 μg of each protein was separated with SDS-polyacrylamide gel electrophoresis (PAGE). After the size-dependent separation, the proteins were transferred to polyvinylidene difluoride (PVDF) membranes using semi-dry transfer (Bio-Rad, Hercules, CA, USA). The membranes were incubated with primary antibodies overnight at 4 °C, followed by incubation with horseradish peroxidase (HRP)-conjugated secondary antibodies (Abcam, Cambridge, UK) for 1 h at room temperature. The signals were detected with chemiluminescence reagents (Abclon). Anti-acetyl histone H3 (Merck Millipore, 06-599, Burlington, MA, USA), anti-histone H3 (Santa Cruz Biotechnology, SC-10809, Dallas, TX, USA), anti-acetylated α tubulin (Santa Cruz Biotechnology, SC-23950), anti-tubulin (Santa Cruz Biotechnology, SC-32293), anti-Runx2 (Abcam, ab23981), and anti-Adiponectin (Cell Signaling Technology, #2789, Danvers, MA, USA) were used for the immunoblotting assay in this study. The levels of acetyl H3 and acetylated α tubulin were quantified with ImageJ and normalized to the quantified levels of H3 and α tubulin.

### 2.4. Reverse Transcription and Quantitative Real-Time PCR (RT-qPCR)

The entire RNA was extracted from the cellular samples using the Easy-Blue reagent (Intron Biotechnology, Seongnam, Korea). Then, 1 μg of extracted RNA was reverse transcribed into cDNA using a Maxim RT-PreMix Kit (Intron Biotechnology). Quantitative real-time PCR (qPCR) was performed by mixing cDNA, KAPA SYBR^®^ FAST qPCR Master Mix (Kapa Biosystems, Wilmington, MA, USA), and each primer below. The qPCR reaction was detected by a CFX96 Touch^TM^ or Chromo4^TM^ real-time PCR detector (Bio-Rad, Hercules, CA, USA). The relative mRNA levels were normalized to the levels of *β-actin* mRNA for each reaction. The qPCR primer sequences used are as follow:

*β-actin* forward, 5′-ACGGCCAGGTCATCACTATTG-3′;

*β-actin* reverse, 5′-TGGATGCCACAGGATTCCA-3′;

*Wnt6* forward, 5′-GCGGAGACGATGTGGACTTC-3′;

*Wnt6* reverse, 5′-ATGCACGGATATCTCCACGG-3′;

*Wnt10a* forward, 5′-CCACTCCGACCTGGTCTACTTTG-3′;

*Wnt10a* reverse, 5′-TGCTGCTCTTATTGCACAGGC-3′;

*Wnt10b* forward, 5′-GCTGACTGACTCGCCCACCG-3′;

*Wnt10b* reverse, 5′-AAGCACACGGTGTTGGCCGT-3′;

*Bmp2* forward, 5′-TGGAAGTGGCCCATTTAGAG-3′;

*Bmp2* reverse, 5′-TGACGCTTTTCTCGTTTGTG-3′;

*Ocn* forward, 5′-AGGGCAATAAGGTAGTGAA-3′;

*Ocn* reverse, 5′-GAGGCTCTGAGAAGCATAAA-3′;

*Sp7* forward, 5′-CCCTTCTCAAGCACCAATGG-3′;

*Sp7* reverse, 5′-AAGGGTGGGTAGTCATTTGCATA-3′;

*Cebpa* forward, 5′-CTCCCAGAGGACCAATGAAA-3′;

*Cebpa* reverse, 5′-AAGTCTTAGCCGGAGGAAGC-3′;

*PPARγ* forward, 5′-GCATGGTGCCTTCGCTGA-3′;

*PPARγ* reverse, 5′-TGGCATCTCTGTGTCAACCATG-3′;

*Adipsin* forward, 5′-CATGCTCGGCCCTACATG-3′;

*Adipsin* reverse, 5′-CACAGAGTCGTCATCCGTCAC-3′;

*Fabp4* forward, 5′-AAGGTGAAGAGCATCATAACCCT-3′;

*Fabp4* reverse, 5′-TCACGCCTTTCATAACACATTCC-3′;

*Adipoq* forward, 5′-TGTTCCTCTTAATCCTGCCCA-3′;

*Adipoq* reverse, 5′-CCAACCTGCACAAGTTCCCTT-3′;

*Leptin* forward, 5′-GAGACCCCTGTGTCGGTT-3′;

*Leptin* reverse, 5′-CTGCGTGTGTGAAATGTCATTG-3′;

*ATGL* forward, 5′-TTCACCATCCGCTTGTTGGAG-3′;

*ATGL* reverse, 5′-AGATGGTCACCCAATTTCCTC-3′;

*MCAD* forward, 5′-ACCCTGTGGAGAAGCTGATG-3′;

*MCAD* reverse, 5′-AGCAACAGTGCTTGGAGCTT-3′;

*IL6* forward, 5′-TTATATCCAGTTTGGTAGCATCCAT-3′;

*IL6* reverse, 5′-AGGCTTAATTACACATGTTCTCTGG-3′;

*TNFα* forward, 5′-CATCTTCTCAAAATTCGAGTGACAA-3′;

*TNFα* reverse, 5′-TGGGAGTAGACAAGGTACAACCC-3′.

### 2.5. Chromatin Immunoprecipitation and Real-Time PCR (ChIP-qPCR)

Chromatin immunoprecipitation was performed as previously described [19]. In brief, chromatin and protein were cross-linked by 1% formaldehyde, followed by shearing. A small portion (5%) of the chromatin solution was reserved as the input DNA, and the remainder was incubated with each primary antibody and protein A agarose/salmon sperm DNA (Millipore, #16-157) overnight at 4 °C. After immunoprecipitation, chromatin fragments were de-crossed from the proteins and eluted to be subjected to qPCR using the primer pairs specific for the target gene promoter. The primer sequences used for ChIP-qPCR were as follows:

*Wnt6* forward, 5′-CTTCCTTCCCCCAAAGAAATG-3′;

*Wnt6* reverse, 5′-GTCCAACAGCTCTTCCCTACCTATC-3′;

*Wnt10a* forward, 5′-TCCTTCCTTCACCCTCTGCAT-3′;

*Wnt10a* reverse, 5′-TAGTGTCTAAGGGTTCTACCCCAAGT-3′;

*Wnt10b* forward, 5′-TCCAAAGGAAAGGTTTTAGCCATA-3′;

*Wnt10b* reverse, 5′-CCCACCCTTCCTGCTGAA -3′;

*Runx2* forward, 5′-AGGCCTTACCACAAGCCTTT-3′;

*Runx2* reverse, 5′-TGGAGAGGCAGAATCATGTG-3′.

### 2.6. Oil-Red-O Staining

The lipid droplets accumulated in the adipocytes were visualized using oil-red-O staining. The fully differentiated adipocytes were fixed by incubation with 10% formaldehyde for 1 h and washed with 60% isopropanol. Then, the cells were incubated with the Oil-red-O working solution for 1 h, followed by washing with distilled water three times. To prepare the Oil-red-O stock solution, 300 mg of Oil-red-O powder was dissolved in 100 mL of 99% isopropanol. The Oil-red-O working solution was prepared just before use by diluting 30 mL of the stock solution with 20 mL of distilled water.

### 2.7. Statistical Analysis

The statistical significance was determined through Student’s *t*-test (two-tailed) with Excel (Microsoft, Redmond, WA, USA) and assessed based on the resulting *p*-value.

## 3. Results

### 3.1. Vulpinic Acid Modulates Acetylation of Histone H3 and α Tubulin Depending on Cell Stage

**Figure 1:**
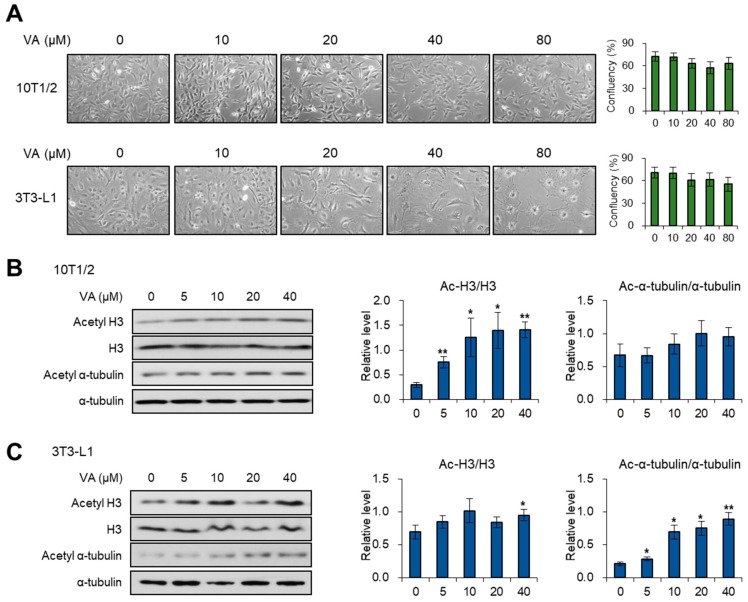
Vulpinic acid (VA) modulates acetylation of histone H3 and α-tubulin in 10T1/2 and 3T3-L1 cells. (**A**) Microscopic images (100X) of 10T1/2 and 3T3-L1 cells treated with vulpinic acid for 24 h at the indicated concentrations. (**B**) Immunoblot analysis of 10T1/2 cells treated with vulpinic acid for 24 h at the indicated concentrations. (**C**) Immunoblot analysis of 3T3-L1 cells treated with vulpinic acid for 24 h at the indicated concentrations. Data represent means ± SEM (standard effort of the mean) for n = 3. * *p* < 0.05, ** *p* < 0.01.

Since previous studies have reported the cytotoxic effects of vulpinic acid to cancer cells [15,16,17], we evaluated the influence of vulpinic acid on the cell death and morphology of 10T1/2 MSCs and 3T3-L1 preadipocytes. The confluency of 10T1/2 and 3T3-L1 cells was slightly reduced at more than 20 μM of vulpinic aicd, but not significantly changed (Figure 1A). In the case of 3T3-L1 cells, there were dramatic morphological changes, becoming a spread pattern with the loss of fibroblastic shape, upon treatment with high concentrations of vulpinic acid (Figure 1A). During early commitment to differentiation, the global acetylation of histone modulates the expression of several key factors [20]. Meanwhile, acetylation of α-tubulin is a prerequisite step for transition from preadipocytes to mature adipocytes [11]. Hence, we assessed the level of acetylated histone H3 after treating 10T1/2 and 3T3-L1 cells with vulpinic acid. In 10T1/2 cells, vulpinic acid treatment enhanced the level of acetylated H3, whereas there was little change in the level of acetylated α-tubulin (Figure 1B). Interestingly, the vulpinic acid-treated 3T3-L1 cells showed a notable increase in only acetylated α-tubulin, without significant alterations in acetyl H3 (Figure 1C). These data indicate that vulpinic acid would lead to the cellular remodeling associated with the differentiation process in the two cell lines.

### 3.2. Vulpinic Acid Promotes the Expression of Wnt Genes via H3 Acetylation in 10T1/2 MSCs

**Figure 2:**
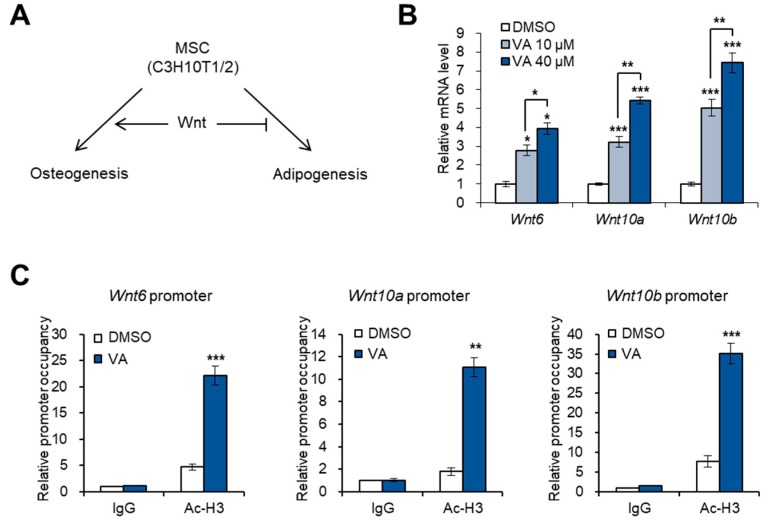
Vulpinic acid promotes the expression of Wnt genes via H3 acetylation in 10T1/2 MSCs. (**A**) Schematic representation of Wnt signaling-mediated cell fate determination of mesenchymal stem cells (MSCs). (**B**) The mRNA levels of Wnt6, Wnt10a, and Wnt10b genes in 10T1/2 cells treated with vulpinic acid (10 or 40 μM) for 24 h. (**C**) 10T1/2 cells were treated with or without vulpinic acid (40 μM) for 24 h. ChIP assay was performed with IgG and acetylated H3 antibodies followed by real time PCR with primers for promoter regions of Wnt6, Wnt10a, and Wnt10b genes. Data represent means ± SEM for n = 3. * *p* < 0.05, ** *p* < 0.01, *** *p* < 0.001.

To estimate the effects of vulpinic acid on the cell fate conversion of 10T1/2 MSCs, we examined the expression of Wnt genes, which are important for the determination of cell lineage during the commitment step of multipotent stem cells [21,22]. Wnt6, 10a, and 10b promote osteogenic commitment, preventing cells from entering the adipogenic commitment [21,22,23] (Figure 2A). Treatment of 10T1/2 cells with vulpinic acid significantly increased the mRNA level of the three Wnt genes (Figure 2B). A recent study showed that an increase in H3 acetylation enhances the transcription of Wnt genes promoting osteogenic differentiation of bone marrow-derived MSCs [23]. As expected, our ChIP-qPCR data showed that the enrichment of acetylated H3 on the promoter region of Wnt6, 10a, and 10b was largely increased upon vulpinic acid treatment (Figure 2C). These data suggest that vulpinic acid can determine the cell fate between osteogenesis and adipogenesis by epigenetically enhancing the expression of key controllers.

### 3.3. Vulpinic Acid Induces Osteogenesis via H3 Acetylation in 10T1/2 MSCs

**Figure 3:**
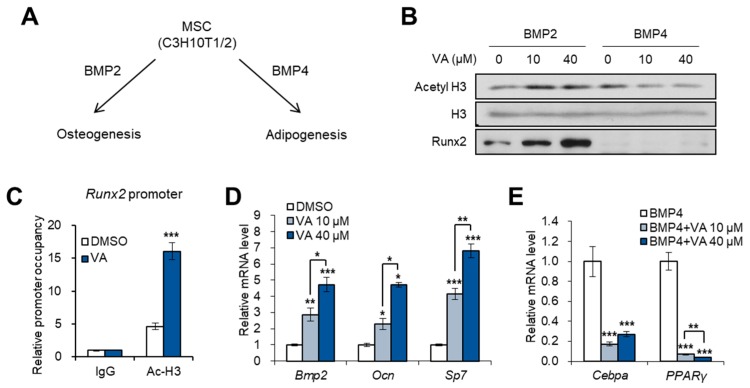
Vulpinic acid induces osteogenesis via H3 acetylation in 10T1/2 MSCs. (**A**) Schematic representation of BMPs-mediated osteogenic or adipogenic commitment of MSCs. (**B**) Immunoblot analysis of 10T1/2 cells treated with vulpinic acid (10 or 40 μM) for 24 h in the presence of BMP2 or BMP4. (**C**) 10T1/2 cells were treated with or without vulpinic acid (40 μM) for 24 h in the presence of BMP2 or BMP4. ChIP assay was performed with IgG and acetylated H3 antibodies followed by real time PCR with primers for promoter region of Runx2 gene. (**D**) The mRNA levels of the *Bmp2*, *Ocn*, and *Sp7* genes in 10T1/2 cells treated with vulpinic acid (10 or 40 μM) for 24 h. (**E**) The mRNA levels of Cebpa and PPARγ genes in 10T1/2 cells treated with vulpinic acid (10 or 40 μM) for 24 h in the presence of BMP4. Data represent means ± SEM for n = 3. * *p* < 0.05, ** *p* < 0.01, *** *p* < 0.001.

Based on our observation that vulpinic acid promotes the expression of Wnt genes, we further investigated whether vulpinic acid enhances osteogenic differentiation of MSCs. Bone morphogenetic proteins (BMPs) can induce the differentiation of MSCs into bone, cartilage, and fat lineages. Several studies have reported that BMP2 exhibits the highest osteogenic potential [24,25], while BMP4 triggers the adipogenic commitment of 10T1/2 cells [26] (Figure 3A). Co-treatment with vulpinic acid and BMP2 elevated the acetylation of H3 and the protein level of Runx2, a osteogenic marker (Figure 3B). However, in the presence of BMP4, vulpinic acid failed to increase both acetyl H3 and Runx2 (Figure 3B). Consistent with an earlier study that show that H3 acetylation are elevated in osteogenic genes during 10T1/2 osteogenesis [27], vulpinic acid induced hyper-acetylation of H3 on the Runx2 gene (Figure 3C). In addition, the transcriptions of other osteogenic marker genes were significantly increased upon vulpinic acid treatment in 10T1/2 cells (Figure 3D). On the contrary, BMP4-mediated expression of adipogenic commitment marker genes, such as Cebpa and PPARγ, were interrupted by vulpinic acid (Figure 3E). These data imply that vulpinic acid promotes osteogenesis and prevents adipogenic commitment in MSCs, coordinating with BMP signaling.

### 3.4. Vulpinic Acid Promotes Adipogenesis from 3T3-L1 Preadipocytes

**Figure 4:**
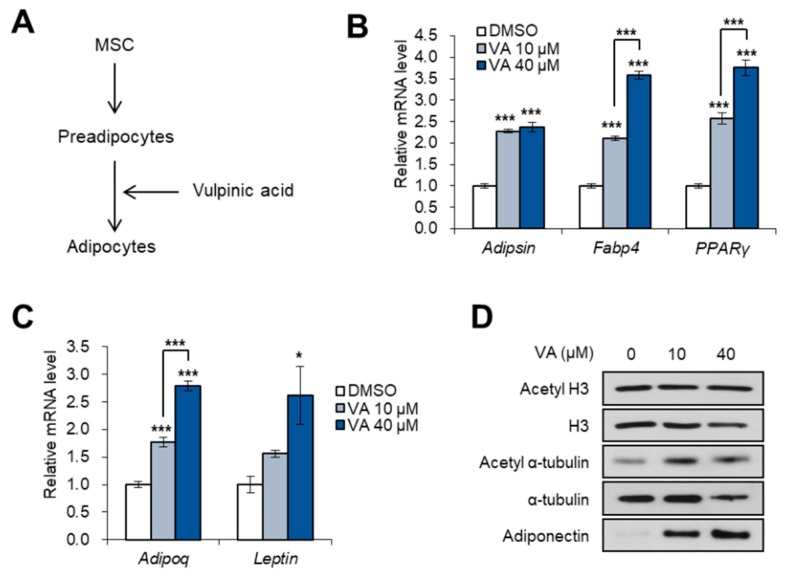
Vulpinic acid promotes adipogenesis from 3T3-L1 preadipocytes. (**A**) Schematic representation of adipogenesis process. 3T3-L1 cells were incubated with an adipogenic medium in the absence or presence of vulpinic acid. (**B**) The mRNA levels of Adipsin, Fabp4, and PPARγ genes in 3T3-L1 adipocytes incubated with vulpinic acid (10 or 40 μM) during adipogenesis. (**C**) The mRNA levels of Adipoq and Leptin genes in 3T3-L1 adipocytes incubated with vulpinic acid (10 or 40 μM) during adipogenesis. (**D**) Immunoblot analysis of 3T3-L1 adipocytes incubated with vulpinic acid (10 or 40 μM) during adipogenesis. Data represent means ± SEM for n = 3. * *p* < 0.05, ** *p* < 0.01, *** *p* < 0.001.

Next, we investigated the effects of vulpinic acid on 3T3-L1 preadipocytes, which already passed the commitment step from MSCs (Figure 4A). Unlike 10T1/2 cells, adipocyte marker genes were highly expressed in mature adipocytes from 3T3-L1 cells which were exposed to vulpinic acid during terminal differentiation (Figure 4B). Moreover, the expressions of adipokines, adipoq and leptin were also elevated in 3T3-L1 adipocytes incubated with vulpinic acid during adipogenesis (Figure 4C), indicating a greater amount of functional adipocytes. The protein level of adiponectin also increased by incubation with an adipogenic medium containing vulpinic acid, which was accompanied by an increase in acetyl α-tubulin without any alterations in acetyl H3 (Figure 4D). Given that cytoskeletal remodeling by microtubule dynamics is required for the differentiation of preadipocytes to mature adipocytes [11], vulpinic acid-mediated elevation of acetyl α-tubulin would contribute to excessive generation of mature adipocytes.

### 3.5. Vulpinic Acid Reduces the Characteristics of Hypertrophic Adipocytes

**Figure 5:**
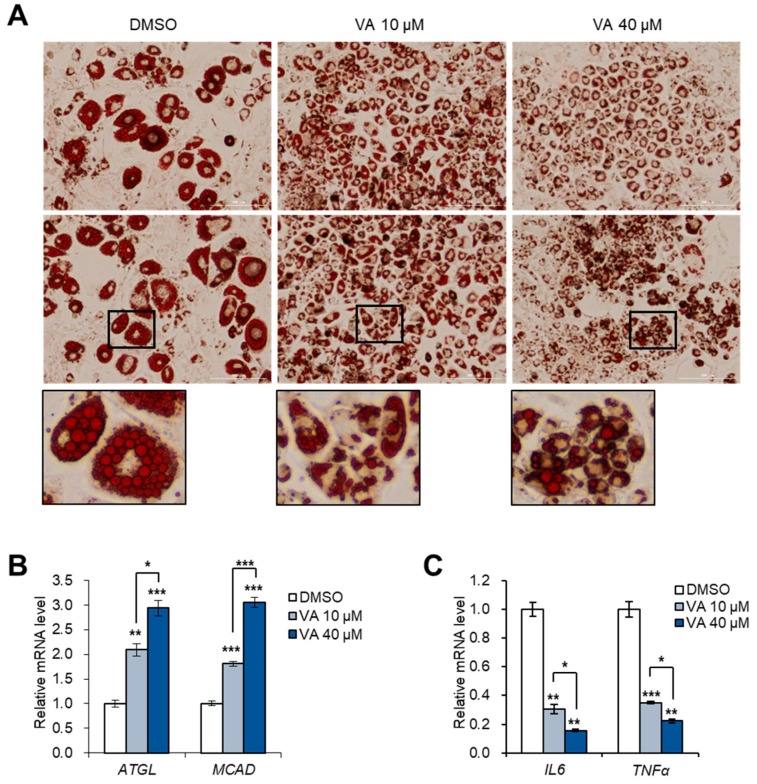
Vulpinic acid reduces hypertrophic adipocytes by promoting hyperplasia. (**A**) Oil-red-O staining of 3T3-L1 adipocytes incubated with vulpinic acid (10 or 40 μM) during adipogenesis. Scale bar = 200 μm (40X). (**B**) The mRNA levels of ATGL and MCAD genes in 3T3-L1 adipocytes incubated with vulpinic acid (10 or 40 μM) during adipogenesis. (**C**) The mRNA levels of IL6 and TNFα genes in 3T3-L1 adipocytes incubated with vulpinic acid (10 or 40 μM) during adipogenesis. Data represent means ± SEM for n = 3. * *p* < 0.05, ** *p* < 0.01, *** *p* < 0.001.

An overload of lipids in a limited number of adipocytes leads to the inappropriate hypertrophy of adipocytes, which is commonly observed in fat depots of obese adults [28]. In line with our PCR data showing that vulpinic acid largely boosted adipogenic capacity of 3T3-L1 cells (Figure 4), vulpinic acid prevented the enlargement of adipocytes dispersing lipid drops across multiple adipocytes (Figure 5A). The mRNA expressions of lipolytic genes, ATGL and MCAD, were enhanced upon treatment with vulpinic acid (Figure 5B), demonstrating the suppressed lipid accumulation which was detected by oil-red-O staining (Figure 5A). Abnormally hypertrophic adipocytes induce inflammation, which is closely associated with adipose tissue dysfunction and impaired insulin sensitivity in obesity-mediated diabetes [29]. Treatment with vulpinic acid significantly reduced the expression of inflammatory marker genes (Figure 5C), implying that vulpinic acid has the effect of alleviating the hypertrophic characterizations of adipocytes by promoting the differentiation of adipocyte precursor cells.

## 4. Discussion

Mushrooms are emerging as promising sources for naturally occurring novel bioactive compounds, as many studies have found their bioactive properties, including anti-cancer, anti-diabetic, anti-obesity, and hypocholesterolemic effects [30]. These beneficial effects of mushrooms on human health are attributed to a large number of secondary metabolites in their extracts. A variety of investigations into mushrooms have demonstrated that mushroom extracts or their metabolites, such as polysaccharides, oligopeptides, polyphenols, and fibers, significantly reduce hypertension, atherosclerosis, lipid accumulation, and obesity [31,32,33]. In addition to these previous works, we here identified the efficacy of vulpinic acid, a mushroom-derived metabolite from *P. ravenelii*, on the formation of adipocytes and osteoblasts. The treatment of mesenchymal stem cells with vulpinic acid led to a remarkable increase in the expression of osteogenic lineage marker genes, interrupting the expression of adipogenic transcription factors (Figure 3). These effects of vulpinic acid on cell fate control resulted from the induction of three Wnt genes, Wnt6, 10a, and 10b, which are well known to inhibit adipogenesis and osteoblastogenesis [22]. Considering the fact that the functions of three Wnt ligands were strongly dependent on a β-catenin [22], β-catenin could be putatively involved in vulpinic acid-mediated regulation of lineage determination. Intriguingly, exposure to vulpinic acid during terminal differentiation of 3T3-L1 preadipocytes rather promoted adipogenesis (Figure 4), inducing hyperplasia of the adipocyte population, which subsequently diminished hypertrophic features of adipocytes (Figure 5).

Inhibiting adipogenesis has been considered an effective way of ameliorating obesity, based on the idea of reducing fat storage space. However, this conventional method might fail to cure adult obesity, because the number of adipocytes is determined early in life and is mostly stable in adulthood [28,34]. Hypertrophic obesity with an increase in adipocyte size has been associated with deleterious effects on dyslipidemia [35], insulin resistance [36,37], and type 2 diabetes [38,39]. On the contrary, the hyperplasia of adipocytes is implicated in protective effects against such metabolic disturbances [40,41]. Thus, recent studies have highlighted the importance of healthy adipocyte generation to preserve systemic metabolic health in obese individuals [42]. In this current study, we found that treatment with vulpinic acid during the maturation of preadipocytes promoted the hyperplasia of functional adipocytes expressing a high level of adiponectin, which is an adipokine secreted from small size of adipocytes (Figure 4). This hyperplastic adipocyte expansion prevented the enlargement of adipocytes with promoted expression of lipogenic genes and reduced expression of inflammatory factors (Figure 5), raising the possibility of using vulpinic acid to manage metabolic disorders.

In the early stages of the terminal differentiation from preadipocytes, the acetylation of α-tubulin increases, mediating the cytoskeletal remodeling and size modification of primary cilium [11,43]. Primary cilium elongation is essential for adipogenesis, and the deacetylation of α-tubulin leads to the disappearance of cilium, subsequently impairing the generation of adipocytes [43,44]. Vulpinic acid largely increased the level of acetyl α-tubulin in 3T3-L1 preadipocytes only, whereas treatment on 10T1/2 MSCs did not have much effect on acetylation of α-tubulin (Figure 1). Long-term treatment with vulpinic acid during the whole process of terminal differentiation from 3T3-L1 cells also elevated the acetylation of α-tubulin (Figure 4D). These data provide better understanding of the way vulpinic acid advances adipogenesis only in 3T3-L1 not in 10T1/2 cells.

Another important issue we present here is that vulpinic acid alters the epigenetic program of osteogenic genes. Osteogenesis is finely controlled through a dynamic orchestration of active and repressive histone marks [45]. The decline of acetylated H3 on *Wnt* genes is responsible for the defect of osteogenesis during osteoporosis [23]. Thus, our observations suggest that vulpinic acid would be helpful in recovering the osteogenic capacities of MSCs in osteoporosis patients. Several more recent studies have reported the osteogenic effects of HDAC inhibitors [46,47,48]. In our data, the treatment of 10T1/2 MSCs with vulpinic acid increased H3 acetylation, both at the global level and at the local level, on the promoter regions of Wnt genes and the Runx2 gene. Considering its significant influence on histone acetylation and subsequent gene activation, our findings suggest vulpinic acid to be a potential epigenetic modulator and provide a molecular mechanism of action underlying its osteogenic activity. Further genome-wide analysis would help to understand the more extensive effects of vulpinic acid on the epigenetic gene regulation.

## 5. Conclusions

**Figure 6:**
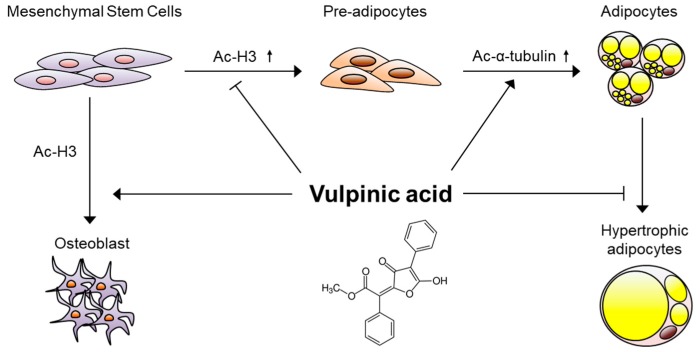
Molecular model underlying the mechanism of actions of vulpinic acid. During the early stage of mesenchymal stem cells, vulpinic acid promotes osteogenic commitment while preventing adipogenic commitment via H3 acetylation-mediated gene modulations. During terminal differentiation to mature adipocytes from preadipocytes, vulpinic acid enhances de novo generation of adipocytes via acetylation of α-tubulin, reducing hypertrophic adipocytes.

In this current study, we demonstrate the multiple actions of vulpinic acid on the formation process of cells important for metabolism (Figure 6). Lichens containing vulpinic acid have historically been used to poison predators [13], and thus studies about the pharmacological influence of vulpinic acid have exploited their cytotoxicity [13,14,15,16,17]. Our findings suggest mushroom-derived vulpinic acid as a promising therapeutic option to improve symptoms of obesity and related metabolic diseases.
